# Role of Synthetic Plant Extracts on the Production of Silver-Derived Nanoparticles

**DOI:** 10.3390/plants10081671

**Published:** 2021-08-13

**Authors:** Sabah Al-Zahrani, Sergio Astudillo-Calderón, Beatriz Pintos, Elena Pérez-Urria, José Antonio Manzanera, Luisa Martín, Arancha Gomez-Garay

**Affiliations:** 1 Research Group FiVe-A, Plant Physiology Unit, Faculty of Biology, Universidad Complutense de Madrid, Ciudad Universitaria, 28040 Madrid, Spain; sabahalz@ucm.es (S.A.-Z.); serastud@ucm.es (S.A.-C.); bpintos@ucm.es (B.P.); elenapuc@ucm.es (E.P.-U.); lmartin@ucm.es (L.M.); 2 Research Group FiVe-A, College of Forestry and Natural Environment, Universidad Politécnica de Madrid, Ciudad Universitaria, 28040 Madrid, Spain; joseantonio.manzanera@upm.es

**Keywords:** silver nanoparticles, plant non-enzymatic antioxidants, micellar structures, cyanide

## Abstract

The main antioxidants present in plant extracts—quercetin, β-carotene, gallic acid, ascorbic acid, hydroxybenzoic acid, caffeic acid, catechin and scopoletin—are able to synthesize silver nanoparticles when reacting with a Ag NO_3_ solution. The UV-visible absorption spectrum recorded with most of the antioxidants shows the characteristic surface plasmon resonance band of silver nanoparticles. Nanoparticles synthesised with ascorbic, hydroxybenzoic, caffeic, and gallic acids and scopoletin are spherical. Nanoparticles synthesised with quercetin are grouped together to form micellar structures. Nanoparticles synthesised by β-carotene, were triangular and polyhedral forms with truncated corners. Pentagonal nanoparticles were synthesized with catechin. We used Fourier-transform infrared spectroscopy to check that the biomolecules coat the synthesised silver nanoparticles. X-ray powder diffractograms showed the presence of silver, AgO, Ag_2_O, Ag_3_O_4_ and Ag_2_O_3_. Rod-like structures were obtained with quercetin and gallic acid and cookie-like structures in the nanoparticles obtained with scopoletin, as a consequence of their reactivity with cyanide. This analysis explained the role played by the various agents responsible for the bio-reduction triggered by nanoparticle synthesis in their shape, size and activity. This will facilitate targeted synthesis and the application of biotechnological techniques to optimise the green synthesis of nanoparticles.

## 1. Introduction

Nanotechnology, science and technology at the nanoscale of atoms and molecules, will help to address major societal challenges such as climate change, reducing carbon emissions, developing renewable energy, using resources more efficiently and meeting the medical needs of an ageing population [1].

In recent years there has been growing interest in the search for new strategies for synthesising nanoparticles to minimise waste and achieve more sustainable and environmentally friendly processes. The use of substances of biological origin, environmentally friendly solvents and renewable materials has become the key to the development of green synthesis of nanoparticles [2]. Biogenic techniques are included in the so-called green technologies because they do not involve the use of toxic chemicals or the high energy expenditure that accompanies chemical or physical synthesis [3]. 

Metallic nanoparticles are of great scientific and technological interest because of their specific properties, which differ from those of conventional bulk materials, making them new technical tools [4]. Among the metallic nanoparticles obtained by green synthesis, those of silver stand out with numerous uses in electronics, clothing, paints, cosmetics, bactericides, biofungicides, biomedical applications, in the medical-pharmaceutical and food industries, etc. [5]. Furthermore, silver oxide nanoparticles have great biomedical applications [6]. In particular, silver (I) oxide shows great promise for use in medical polymers and nanodrugs [7].

Different biological taxa (mainly plants, fungi and bacteria) have demonstrated their capacity to transform metallic ions into metallic nanoparticles thanks to the reducing ability of the proteins and metabolites present in these organisms. Among them, plants allow a fast, non-pathogenic and cheap synthesis in a single step thanks to the presence of phytochemicals that act as reducing agents in the synthesis of metallic nanoparticles [8].

Many plant species allow the synthesis of silver nanoparticles (AgNPs). There are numerous publications in recent years in which the synthesis of AgNPs using bioorganic compounds is referred to (see, for example, the reviews [3,5,9,10,11,12]). Plant extracts have become an excellent resource because of their availability, easy procurement and ability to produce non-toxic nanoparticles safely and economically (e.g., [8]).

Plants have the ability to reduce metal ions on their surface or absorb them through the roots and transport them to various organs and tissues where reduction can also occur. Plant extracts can act on AgNPs synthesis by reducing Ag^+^ ions. The nanoparticle synthesis capacity of a plant extract depends on the active compound responsible for ion reduction from Ag^+^ to Ag^0^ and varies depending on the organism or plant extract used [13].

Plants have an innate ability to biosynthesise a wide range of antioxidants capable of mitigating oxidative damage induced by reactive oxygen species (ROS). These reducing agents consist of a large number of organic molecules such as carbohydrates, fats, proteins, enzymes, coenzymes, phenols, flavonoids, terpenes, alkaloids, etc. [14], which are capable of producing silver nanoparticles from, for example, the reduction of silver nitrate, AgNO_3_ [15].

In plant cells, there are three organelles in which oxidative stress is mainly produced with the generation of ROS. ROS are generated in photosystems I and II in chloroplasts, in ubiquinone and complex III in the mitochondrial electron transport chain and, to a lesser extent, in the peroxisome membrane and matrix [16]. Most of the time the production of these free radicals is regulated because they act as signalling molecules. The control of these radicals is highly efficiently based on two complementary systems: the enzymatic and the non-enzymatic [17]. The enzymatic system comprises various enzymes (superoxide dismutase, catalase, glutathione peroxidase and glutathione reductase) which are highly specific to the substrate. The non-enzymatic control system comprises low molecular weight antioxidants (ascorbic acid, glutathione, proline, carotenoids, phenolic acids, flavonoids, etc.) and secondary high molecular weight metabolites, such as tannins, which act as free radical scavengers, reducing agents and metal chelators.

Many plant families and species synthesize hundreds of unique phytochemical substances [18]. Around 200,000 compounds have been described in approximately 30% of higher plants, and many taxa remain unexplored. This diversity is further amplified by plasticity, as phytochemistry varies with ontogeny and phylogeny in response to abiotic factors and biotic interactions [19,20,21]. Also, phytochemical diversity varies between branches, organs, and even tissues within organs [22,23]. As sessile organisms, plants have adapted to their environment by synthesizing an enormous variety of low-molecular-weight secondary metabolites, with ecological and evolutionary functions [24].

However, secondary metabolites are restricted to a few biosynthetic pathways derived from the primary pathways, i.e., glycolysis, the Krebs cycle or the shikimic acid pathway. Nevertheless, the enzymes are highly specific, implying that the final products almost always have different stoichiometry. This secondary biosynthetic process is highly regulated and coordinated, involving different cell types, transport routes and cell compartments [25].

Usually, secondary metabolites are genetically regulated and synthesized in specific organs, tissues, cells or organelles, and this specificity is regulated throughout plant development. Therefore, numerous transcription factors are coordinated for the activation and transcription of genes. The cytoplasm is an usual site of synthesis but some alkaloids, coumarins and terpenes, for example, are synthesized in the chloroplast, or some sesquiterpenes and sterols are synthesized in the endoplasmic reticulum [26]. Water-soluble compounds are transported and stored in the vacuole by specific transporters, through the tonoplast. Lipophilic substances are confined in resin ducts, laticifers, glands, trichomes, thylakoid membranes or in the cuticle [26], avoiding autotoxicity by interference with biomembranes.

In many cases, the site of biosynthesis of certain compounds is limited to a single organ, such as the root, the leaf or the fruit, but later is transported to and accumulated in other plant tissues. In these cases, long-distance transport occurs through the xylem or phloem, although apoplastic transport may also be possible. This storage may be specific to a given tissue or cell, e.g., tannins, alkaloids or glucosinolates are stored in idioblasts; terpenoids in trichomes or glandular hairs; flavonoids, antocyanins or cyanogenic glycosides in the epidermis. The metabolic profile of a given plant species, its content in phytochemical compounds and particularly in secondary metabolites, varies with time, space and stage of development [27].

Vitamin C (ascorbic acid/ascorbate) is generated in aerobic metabolism and easily donates an electron to potentially harmful oxidising radicals such as hydroxyl, radical (HO^−^), alkoxyl radical (RO^−^), peroxyl radical (ROO^−^), thiol radical (RS^−^) and tocopheroxyl radicals [28]. 

β-Carotene is the most common carotenoid in plants. It is a fat-soluble pigment with antioxidant activity. As an antioxidant its activity is produced on singlet oxygen and other electronically excited molecules that are generated in photo or chemo excitation reactions. It also reacts with the alkoxyl (RO^−^) and peroxyl (ROO^−^) radicals [29]. 

**Figure 1:**
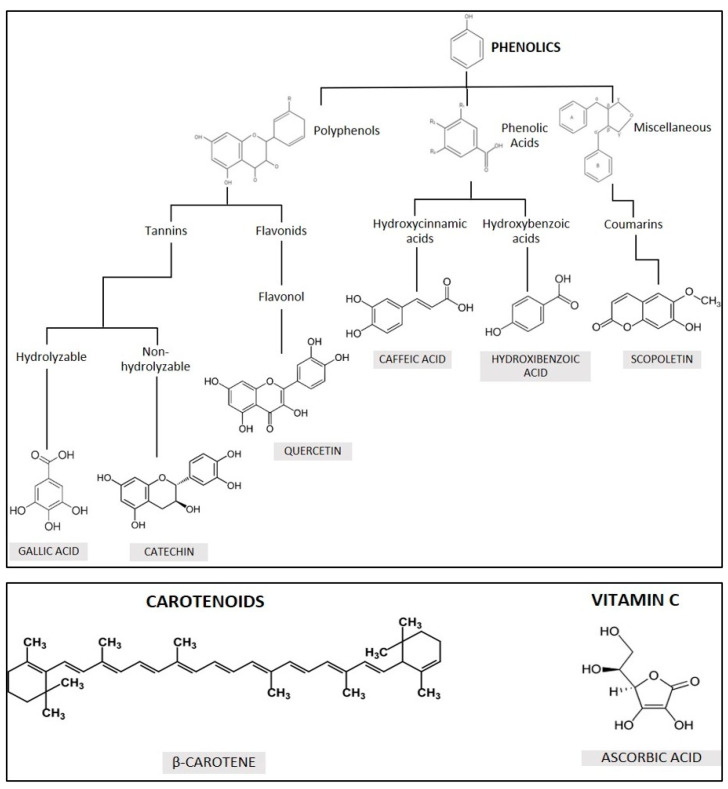
Natural non-enzymatic antioxidants present in plants and used in this work.

Phenolic compounds are classified into five main groups (Figure 1): tannins, flavonoids, hydroxycinnamic acids, hydroxybenzoic acids and others [30]. Phenolic compounds have in common the presence of a phenol in their structure. Depending on the number of phenols, they are classified into: Simple phenols with only one phenol (phenolic acids); Polyphenols with two phenols (flavonoids and stilbenes) or more (tannins). 

Phenolic acids have a carboxylic group in two different carbon structures, resulting in structures derived from hydroxycinnamic acid (e.g., caffeic acid) or hydroxybenzoic acid [31]. 

Flavonoids, such as quercetin, are polyphenolic compounds whose characteristic basic structure is the flavone nucleus (2-phenyl-benzo-gamma-pyran) formed by two benzene rings (A and B) joined by a pyrane-ring (C) containing oxygen [32].

Hydrolysable tannins are compounds with a central nucleus of glucose or another polyol esterified with gallic acid or with hexahydroxydiphenic acid [33]. Hydrolysable tannins are present only in dicotyledonous angiosperms. Condensed tannins, also called proanthocyanidins, are oligomers or polymers with a flavan-3-ol core. Both hydrolysable tannins (such as gallic acid) and condensates (such as catechin) remove the activity of the superoxide radical, hydroxyl (HO^−^), peroxyl radical (ROO^−^) and NO [34].

Coumarins are compounds with a benzene ring condensed to a lactone. All coumarins exhibit antioxidant activity. Scopoletin, in particular, exhibits significant antioxidant activity [35]. 

Despite the many advantages of plant extracts for the synthesis of AgNPs, there are also disadvantages. Particle size and morphology are difficult to control. The use of these nanoparticles still has limitations mainly due to the low efficiency of the synthesis methods and the lack of size standardization [36]. In addition, the variability and functionality of plant extracts is often unknown, which makes it difficult to understand the formation mechanisms compared to established chemical approaches [37]. The differences in the agents responsible for the bio-reduction triggered by nanoparticle synthesis result in the production of nanoparticles with different shapes, sizes and bioactivity. 

The IR spectra provide information about the organic molecules on the surface of nanoparticles. Fourier transform infrared spectroscopy (FTIR) analysis is carried out to identify the possible biomolecules responsible for capping the silver nanoparticles. Thus, diverse spectra were obtained depending on the reduced agent implied in the nanoparticle synthesis. Furthermore, XRD (X-ray diffraction) has a good potential for the analysis of nanoparticles, because the width and shape of reflections yield information about the substructure of the materials. XRD analysis is useful to determine the crystal structure and calculate the crystalline nanoparticles size. The Scherrer method is the most widely applied approach to analysis of the X-ray diffraction line profiles. Silver metal has a crystal structure of face-centred cubic (fcc) lattice. Bragg reflections for silver nanoparticles (Ag^0^) correspond to the (111), (200), (220), (311) and (222) reflections of fcc silver.

Achieving large-scale, cost-effective production of nanoparticles requires research related to the isolation and purification of plant metabolites responsible for their synthesis, thus facilitating the application of genetic and metabolic engineering techniques [3].

In light of these considerations, the main objective of this work is to study in detail the synthesis of silver nanoparticles by the main non-enzymatic antioxidants present in plants (Figure 1) in order to determine their role in the process of green nanoparticle synthesis by means of plant extracts.

## 2. Results and Discussion

**Figure 2:**
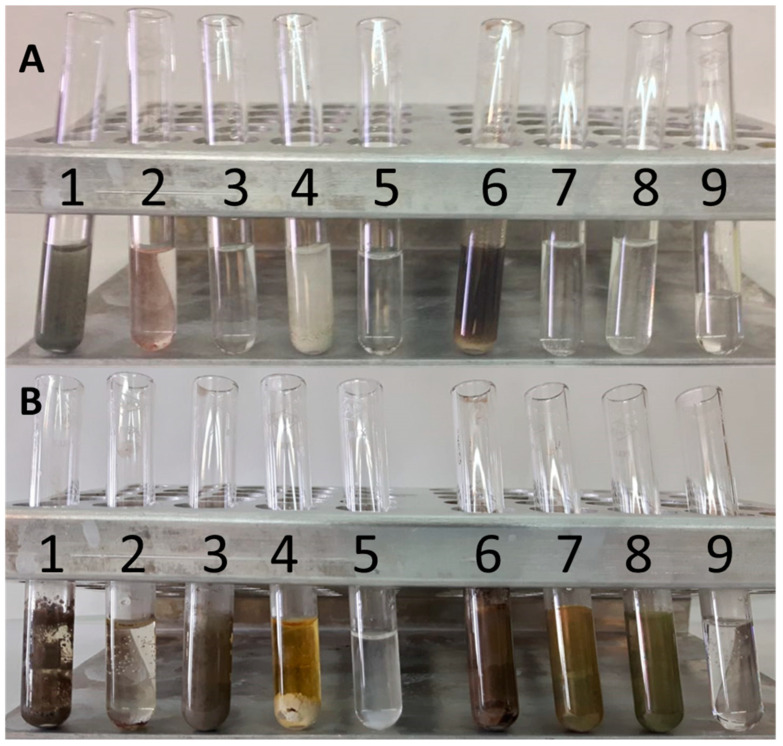
Colour changes after the addition of different antioxidants of plant origin to a 2 M silver nitrate solution. (**A**) inception (time 0); (**B**) after 5 h of incubation at 40 °C in the dark. The numbers identify the different antioxidants used: 1 = quercetin; 2 = β-carotene; 3 = gallic acid; 4 = ascorbic acid; 5 = hydroxybenzoic acid; 6 = caffeic acid; 7 = catechin; 8 = scopoletin; and 9 = control (no antioxidant added).

The addition of the different plant antioxidants to the aqueous silver nitrate solution resulted in a staining of the solution after 5 h of reaction, indicating the formation of silver nanoparticles (Figure 2). The nanoparticles were characterized by spectroscopy absorption, TEM analysis, FTIR analysis, XRD analysis and reactivity of the silver nanoparticles. Although both Ag^0^ silver nanoparticles and silver oxide nanoparticles have been detected, all of them will be mentioned as AgNPs in this article, from now on.

### 2.1. Synthesis of Silver Nanoparticles by Quercetin

Quercetin (3,3′,4′,5,7-pentahydroxyflavone) is a very abundant flavonoid in plants containing five hydroxyl groups in positions 3, 5, 7, 3′, 4′ and one carbonyl group in position four (Figure 1). Quercetin has low water solubility. Although it is considered a hydrophobic compound, among flavonoids it has one of the lowest octanol-water partition coefficients (log P = 1.82 ± 0.32; [38]) which implies that its partition in hydrophobic solvents is also limited. This flavonoid has an important role in the elimination of free radicals and also has the ability to easily form complexes with many metals [39]. Due to this last characteristic, quercetin presents a strong interaction with metallic nanoparticles. In addition, quercetin reduces silver ions in aqueous solutions of silver salts (as in this case silver nitrate), probably through the formation of an intermediate complex where the electron density is displaced towards the silver ion [40].

**Figure 3:**
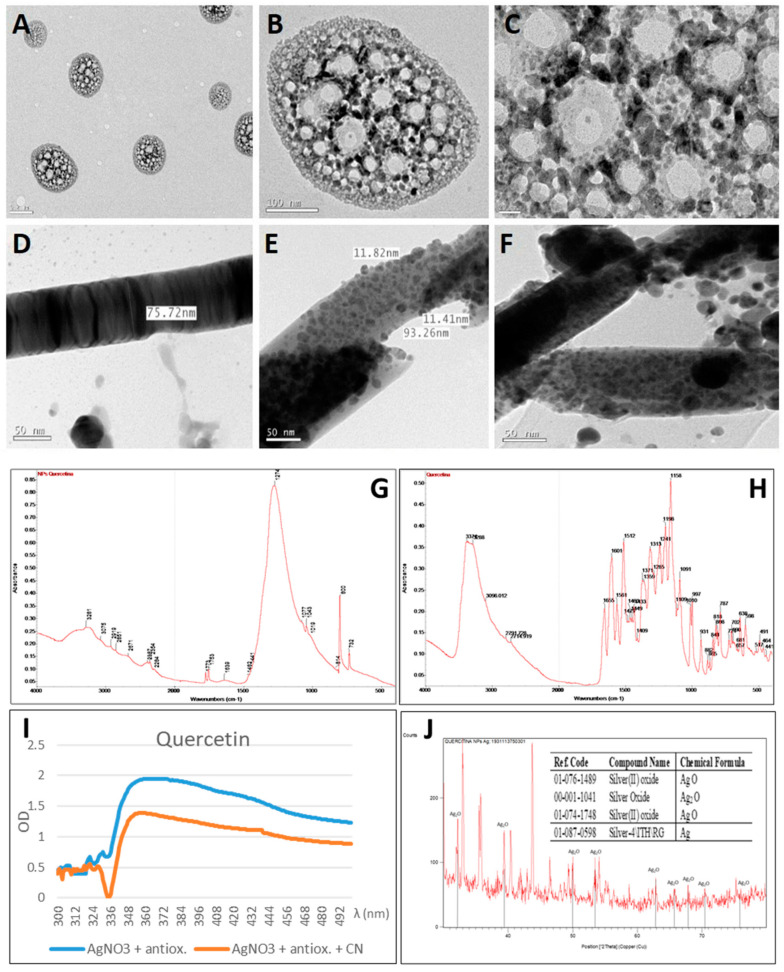
Analysis of quercetin-synthesised silver nanoparticles (AgNP). (**A**–**C**) images of AgNPs obtained by transmission electron microscopy (TEM). (**A**) Micelle-like structures. (**B**) Micellar structure detail. (**C**) Detail of vesicles located between the silver nanoparticles. (**D**–**F**) TEM images of AgNPs after cyanide addition. (**D**) rod-like structure. (**E**) Detail of filament showing the aggregation of the nanoparticles. (**F**) Detail of rod-like structures with their size diameter and comparison with the size of the nanoparticles. (**G**) Fourier transform infrared (FTIR) spectrum of quercetin-derived nanoparticles. (**H**) FTIR spectrum of quercetin. (**I**) Ultraviolet (UV)-visible AgNP spectra prepared with quercetin and AgNO_3_ prior (blue line) and after (orange line) cyanide addition. (**J**) X-ray diffraction (XRD) profile of AgNPs obtained by quercetin reaction with AgNO_3_ (an identification table has been inserted). Peaks identifying the presence of silver oxides have been indicated in the XRD diffractogram.

The change in coloration of the silver nitrate solution caused by quercetin to a greyish colour (Figure 2(A-1,B-1)) is characteristic of added silver nanoparticles. Quercetin reacted immediately with the silver nitrate solution (Figure 2(A-1)) to produce a greyish precipitate characteristic of the added silver nanoparticles that adhered to the walls of the vessel. The study of the UV-visible spectrum (Figure 3I) did not show a surface plasmon band (SPB) characteristic of the surface plasmon excitation of the metallic nanoparticles. Nevertheless, this difference might arise from the method of synthesis. Quercetin mediated synthesis of silver nanoparticles [41,42] and silver nanoparticles in reverse micelles [39] were obtained in the presence of NaOH and by the injection of water salt solution in octane or heptane. The spectrum obtained was clearly different from the known spectra of silver nanoparticles and was due to the formation of large quercetin aggregates [43]. The quercetin molecule suffers chemical modifications at neutral pH, involving the dimerization or oligomerization of the flavonoid on the surface of the AgNPs with an important role of A- and C-rings while, at alkaline pH, the B-ring is responsible of a change in this dimerization protocol [44].

However, analysis by electron microscopy (TEM) allowed the observation of micelle-like structures composed of nanoparticles of various sizes (Figure 3A–C). These structures are characteristic of elements with an amphipathic character in which the polar end is in contact with the aqueous solution while the hydrophobic end is located towards the interior of the micelle. Hydrophobic quercetin would be confined to the vesicles formed between the nanoparticles while smaller nanoparticles are located in the outermost layer of these structures. As the nanoparticles were formed by the reduction caused by quercetin, this in turn was transformed into the products of its oxidation (mainly 3,4-dihydroxy-benzoic and 2,4,6-trihydroxybenzoic acids; [45]). These two acids have a greater hydrophilic nature than quercetin, thus determining the location of the structures of these smaller and more reactive nanoparticles on the surface. 

The association of quercetin with silver nanoparticles was confirmed by FTIR. Figure 3G shows the FTIR spectrum of the silver nanoparticles, while Figure 3H shows the spectrum of quercetin. In the FTIR spectrum of quercetin synthesised silver nanoparticles, four bands in 3281, 1753, 1274 and 800 cm^−1^ stand out. The band at 3281 cm^−1^ indicated the presence of O–H stretching. In the quercetin spectrum, a wide absorption band between 3288 and 3374 cm^−1^ was observed, which also corresponded to O–H stretching. The weak band at 1753 cm^−1^ corresponded to C=O anhydride. The band in the quercetin spectrum at 1158 cm^−1^ corresponded to C–OH stretching. This functional group was modified in the synthesized silver nanoparticles and corresponded to a very intense band in the FTIR spectrum of the silver nanoparticles at 1274 cm^−1^ (C–O stretching; 1210–1320 cm^−1^). The band at 800 cm^−1^ corresponded to a C–H bending (675–1000 cm^−1^) which corresponded to C–H stretching at 3075 cm^−1^. These bands represent the vibrations of the C–H bending in the aromatic ring. Furthermore, a broad band at the range of 500 to 600 cm^−1^ could be attributed to Ag–O vibration [46]. 

The XRD analysis confirmed the synthesis of several silver nanoparticle compounds in different oxidation states: silver (I,III) oxide (AgO), silver oxide (Ag_2_O) and metallic silver (Figure 3J). The pattern with diffraction lines at 2θ values of 38.15°, 44.33°, 64.52° and 77.52° could be indexed to the planes (111), (200), (220) and (311). The XRD analysis confirmed the face-centred cubic structure (FCC). According to Scherrer’s equation, the average crystallite size of the metallic silver crystalline particles was equal to 106 nm.

The reactivity of the nanoparticles analysed by their oxidation in the presence of cyanide showed a decrease in the intensity of the surface plasmon resonance absorption band of the quercetin-synthesised AgNPs (Figure 3I). The resulting nanoparticles observed through TEM (Figure 3D–F) showed how the morphology of the structures was altered. The appearance of rod-like structures was visible. The transformation of silver nanoparticles depending on the environmental conditions is a common phenomenon [47]. Thus, the chemical transformation of silver nanoparticles to AgCN would cause these changes in the size and shape of the nanoparticles and thus change from spherical micelle-like formation to rod-like [48].

### 2.2. Synthesis of Silver Nanoparticles by β-Carotene

β-Carotene is the most common carotenoid, precursor of vitamin A, this orange pigment is mainly found in fruits and vegetables. It belongs to the group of terpenes, actually a tetratherpene of formula C_40_H_56_. Among the carotenes, β-carotene is distinguished by having beta rings at both ends of the molecule. It is considered a lipophilic molecule, with almost no solubility in water (0.0006 g L^−1^ at 25 °C). β-carotene can act as an effective antioxidant [49]. The molecule of β-carotene has the ability to assemble and align 10 metal atoms into complexes in a reversible manner [50]. These characteristics seem to support the role of this molecule in the synthesis of metallic nanoparticles through green synthesis. However, to the best of our knowledge, there is no data on the usefulness of β-carotene in the synthesis of nanoparticles, although it should participate in the green synthesis with plant extracts that are very rich in this compound [51].

**Figure 4:**
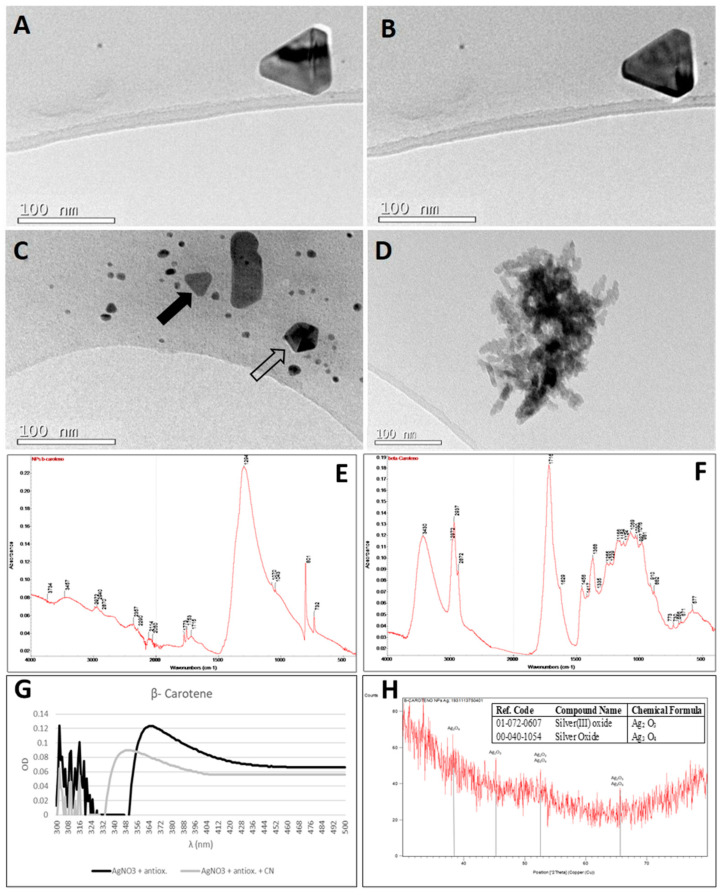
Analysis of the silver nanoparticles synthesised with β-carotene. (**A**–**D**) images of AgNPs obtained by TEM. (**A**,**B**) A triangular nanoparticle under two illumination angles, which allows observing its volume. (**C**) Polyhedral shapes (indicated by the hollow arrow) and triangular shapes with truncated vertexes (indicated by the black arrow). (**D**) Polyhedral Amorphous AgNPs after treatment with cyanide. (**E**) Fourier transform infrared (FTIR) spectrum of β-carotene-derived nanoparticles. (**F**) FTIR spectrum of β-carotene. (**G**) Ultraviolet (UV)-visible AgNP spectra prepared with β-carotene and AgNO_3_ prior (black line) and after (gray line) cyanide addition. (**H**) X-ray diffraction (XRD) profiles of the AgNPs obtained by the reaction of β-carotene with Ag NO_3_; in the insert, the table with the identifications. Peaks identifying the presence of silver oxides have been indicated in the XRD diffractogram.

The colour of the silver nitrate solution after adding the β-carotene was initially orange (Figure 2(A-2)), however, this colouration disappeared, and the solution remained transparent after five hours of reaction at 40 °C (Figure 2(B-2)). At the same time, a slight precipitate appeared at the bottom of the container. When the UV-visible spectrum was analysed (Figure 4G), several SPBs appeared (346 nm, 361 nm; 368 nm and 382 nm) that could be attributed to transverse and longitudinal surface plasmon resonance bands of the anisotropic, triangular and polyhedral silver nanoparticles being formed [52,53,54].

Triangular and polyhedral-shaped nanoparticles (both with the truncated corners) (Figure 4A–C) were observed. Also, spherical nanoparticles of less than 10 nm in diameter and a small triangular particle with the truncated corners of that size (Figure 4C, marked with a black arrow) can be observed. This small triangular particle could be generated from the selective oxidation in different zones of the surface of the spherical nanoparticles (oxidative shape-selective oxidative etching) [55].

For monocrystalline particles, where the whole volume is composed of a single crystalline domain, the thermodynamic equilibrium form is a truncated octahedron (Figure 4C; white arrow) determined by planes/facets 100 and 111. Non-polyhedral forms (triangular in this case, hollow arrow) could be mainly composed of anisotropic structures [56]. The average size of the triangular truncated-corner particles was 60 ± 5 nm (Figure 4A,B). The formation of nanoplates is common in the synthesis of silver and gold nanoparticles [57]. There are two hypotheses proposed to explain the formation of these highly anisotropic structures. The first is the “face-blocking theory”, in which an element selectively adheres to a particular crystalline facet of the growing nanocrystal and therefore slows the growth rate of that facet in relation to the others [58,59]. The second theory explains that preferential anisotropic growth depends on the crystalline symmetry of the initial nuclei [57,60].

The association of β-carotene with silver nanoparticles was confirmed by FTIR. Figure 4E shows the FTIR spectrum of the silver nanoparticles, while Figure 4F shows the spectrum of β-carotene. In the FTIR spectrum of the silver nanoparticles synthesised with β-carotene, five groups of band stand out in 3457, 2972-2940-2870, 1773-1753-1715, 1294 and 801–732 cm^−1^. The band at 3457 cm^−1^ indicated the presence of O–H stretching (alcohol). An absorption band at 3430 cm^−1^ was observed on β-carotene, which also corresponded to this O–H stretching. The band group 2972–2940–2870 cm^−1^ identified the C–H stretching and fall in the spectrum of β-carotene. The band group 1773–1753–1715 cm^−1^ represents the C=O stretching and corresponds to a strong band at 1710 cm^−1^ in the spectrum of β-carotene. The most intense band in the spectrum of the silver nanoparticles synthesized with β-carotene was 1294 cm^−1^ and revealed the C–O stretching. The band at 800 cm^−1^ referred to a C–H bending (675–1000 cm^−1^) which corresponded to the C–H stretching at 2972-2940-2870 cm^−1^. These bands represented the vibrations of the C–H bond in the aromatic ring. Also a broad band between 500–600 cm^−1^ corresponding to metal-oxygen bond has been detected.

The XRD pattern (Figure 4H) indicates that crystalline and amorphous nanoparticles co-exist [61]. In the XRD analysis, different oxidation states, Ag_2_O_3_ and Ag_3_O_4_ silver oxide nanoparticles were identified. An Ag_2_O_3_ and Ag_3_O_4_ mixture was synthesized by using β-carotene. Both Ag_3_O_4_ and Ag_2_O_3_ can also be prepared by anodic oxidation of silver salt solutions at lower potential [62]. The present result is highly relevant, because the industrial production of Ag_2_O_3_ for medical devices and for zinc-mixed alkaline batteries is difficult [7]. Ag_3_O_4_ is more stable than Ag_2_O_3_ [63]. Ag_2_O_3_ presented a cubic structure [64] that corresponded to an octahedral coordination of silver in line with that observed by TEM (Figure 4A–C). The shape of Ag_3_O_4_ was pyramidal [65,66], as observed in Figure 4D [67].

These nanoparticles reacted with cyanide (Figure 4G) and a shift towards shorter wavelengths was observed. The particles resulting from these oxidising conditions did not have defined shapes and were smaller (Figure 4D). The shape of the nanoparticles is decisive in their reactivity [68]. However, in addition, the identified oxides (Ag_2_O_3_ and Ag_3_O_4_) were more catalytically active, possibly more so than the pure metal as traditionally assumed [67]. This high reactivity would be related to the usefulness of triangular silver nanoparticles (especially those with truncated corners) as biosensors [54].

### 2.3. Synthesis of Silver Nanoparticles by Gallic Acid

Gallic acid (gallate; 3,4,5-trihydroxybenzoic acid; 3,4,5-trihydroxybenzoate; pyrogallol-5-carboxylic acid) is a vegetable polyphenol, a hydrolysable tannin, which is a potent antioxidant containing three hydroxyl groups and one acid group. Gallic acid is especially reactive among phenolic compounds probably because of the three carboxyl groups [69]. This organic acid is a natural chelator with a high affinity for metallic compounds [70]. Due to its affinity for metals and its high reactivity, gallic acid has been used in the synthesis of silver nanoparticles (see for example the review of Amini [71]). Gallic acid-AgNPs could be a potential candidate for use in biological and pharmaceutical applications, e.g., to fight microorganism infections [72,73].

**Figure 5:**
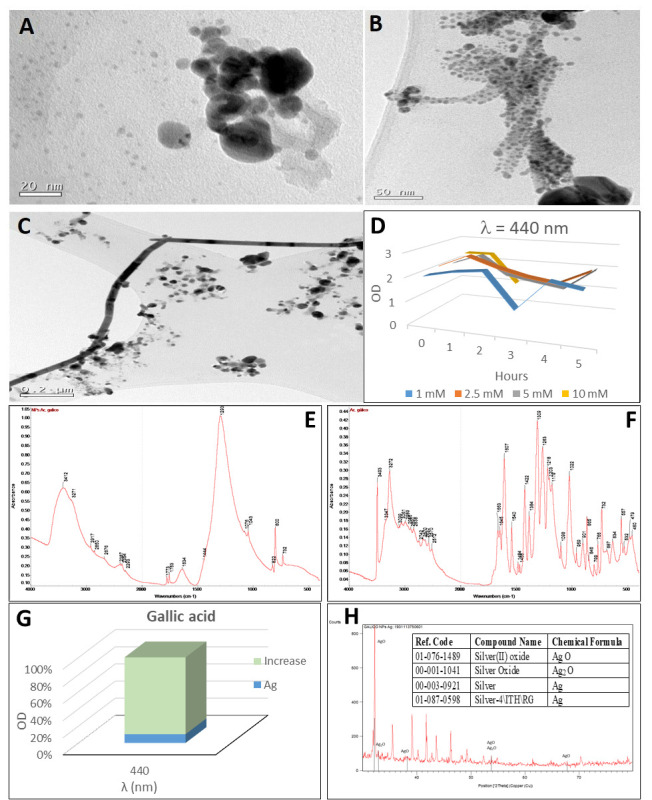
Analysis of silver nanoparticles synthesised with gallic acid. (**A**) TEM image of AgNPs. (**B**,**C**) TEM images of AgNPs after cyanide treatment. (**B**) Aggregation of nanoparticles prior to Figure 3. concentrations with gallic acid for 5 h (at one-hour intervals). (**D**) Synthesis of silver nanoparticles by the reaction of different AgNO_3_ concentrations with gallic acid for 5 h (in one-hour intervals). (**E**,**F**) FTIR spectra of gallic acid-derived nanoparticles (**E**) and gallic acid (**F**). (**G**) Relative increase of OD after cyanide addition to silver nanoparticles. (**H**) XRD diffractogram of the AgNPs obtained by the reaction of gallic acid with Ag NO_3_; in the insert, the table with the identifications. Peaks identifying the presence of silver oxides have been indicated in the XRD diffractogram.

The reaction of gallic acid with silver nitrate produced a characteristic grey colour (Figure 2(A-3)). The reaction was not immediate but took less than an hour (Figure 2(B-3)). It was an intense reaction that provided high optical density values (greater than 2) even with low concentrations of silver nitrate (Figure 5D). The SPB produced a peak at the wavelength of 440 nm which corresponded to a particle size of 70 nm. The concentration of silver nanoparticles of this size was 30 nM (according to Beer–Lambert’s Law) compared to a theoretical molar concentration of 100 nM that would have been obtained if the total conversion of silver ions into silver nanoparticles had taken place (Table 1). 

Gallic acid reduced silver nitrate to silver nanoparticles rapidly at room temperature but aggregates were formed as it did not act as a good stabilizing agent [74]. The formation of these aggregates produced the grey coloration observed in the synthesis of silver nanoparticles with gallic acid.

The presence of aggregates constituted by irregularly shaped nanoparticles with a size close to 20 nm and smaller spherical nanoparticles was observed with the high-resolution transmission electron microscopy (Figure 5A). The size of spherical silver nanoparticles synthesised with gallic acid in chemical synthesis is usually small (10–15 nm [75,76]).

The synthesized silver nanoparticles were coated with gallic acid according to the FTIR spectra (Figure 5E,F). A wide peak was observed at 3412–3271 cm^−1^ identifying the C–H stretching corresponding with the peak at 3272 cm^−1^ for gallic acid. The C=O stretching corresponded to the 1773–1753–1634 cm^−1^ band. An intense peak at 1293 cm^−1^ indicated a C–O stretching which correspond with the peak at 1309 cm^−1^ for gallic acid. The 800 cm^−1^ band identified a C–H bending (675–1000 cm^−1^) which corresponds to the C–H stretching of carboxylic acid in the peaks of 2917–2850–2676 cm^−1^. Between 500–600 cm^−1^ a broad band indicates the presence of silver oxides. The reduction of the silver ions was the result of the oxidation reaction of the phenol groups of gallic acid, and the quinoid compound produced was adsorbed to the surface of the silver nanoparticles, helping to stabilise them [77]. FTIR spectra showed that the carboxylic acid functional groups of gallic acid contributed to the electrostatic bonding on the surface of the nanoparticles [78].

In the XRD analysis (Figure 5G), the presence of silver in different oxidation states: silver (I,III) oxide (AgO), silver oxide (Ag_2_O) and silver was detected. The pattern with diffraction points in 2θ values of 38.15°, 44.33°, 64.52° and 77.52°, that can be indexed to the planes (111), (200), (220) and (311) of the face-centred cubic structure (FCC), is compatible with the Ag cubic phase. The average sizes of the silver crystalline particles were 19 nm for reference code 00-003-0921 and 31 nm for reference code 01-087-0598, as calculated by Scherrer’s equation.

The optical density values of the UV-visible absorption spectrum increased after the addition of cyanide (Figure 5H). These changes in the absorption spectrum could be attributed to the partial oxidation of the silver nanoparticles, with chemical adsorption of Ag^+^ on the particle surface [79]. Since Ag^+^ presents bactericidal properties, these nanoparticles are good candidates for high antimicrobial activity [77]. This characteristic of Ag^+^ ions is due to their high reactivity to cellular components caused by interactions with functional groups such as thiol, carboxylate, phosphate, hydroxyl, imidazole, indole or amine [80]. The presence of oxide on the surface of these AgNPs ensures a high antibacterial activity, most likely due to the higher concentration of ROS they generate [81].

The morphology of the resulting nanoparticles changed to rod-like structures of great length (more than 800 nm), as observed by TEM (Figure 5B,C). The transformation of silver nanoparticles depending on the environmental conditions is a common phenomenon [47]. Again, changes in the size and shape of the nanoparticles from spherical to rod-like form can be observed as a result of the chemical transformation of silver nanoparticles to AgCN [48].

### 2.4. Synthesis of Silver Nanoparticles by Ascorbic Acid

Vitamin C (C_6_H_8_O_6_) in its different forms (ascorbate, ascorbic acid, L-ascorbate, l-ascorbic acid) is a cetolactone with two ionizable hydroxyl groups. Ascorbate mono-anion (AscH^−^) is the predominant form at physiological pH. AscH^−^ can undergo two consecutive oxidations of an electron resulting in the formation of ascorbate (Asc^•−^) and dehydroascorbic acid (DHA) radicals. The ascorbate radical is very slightly reactive and quickly passes to ascorbate and dehydroascorbic acid:

2Asc^•−^ + H^+^→ AscH^−^+ DHA

Ascorbate undergoes pH-dependent auto-oxidation producing hydrogen peroxide. The presence of catalytic metals accelerates this oxidation.

**Figure 6:**
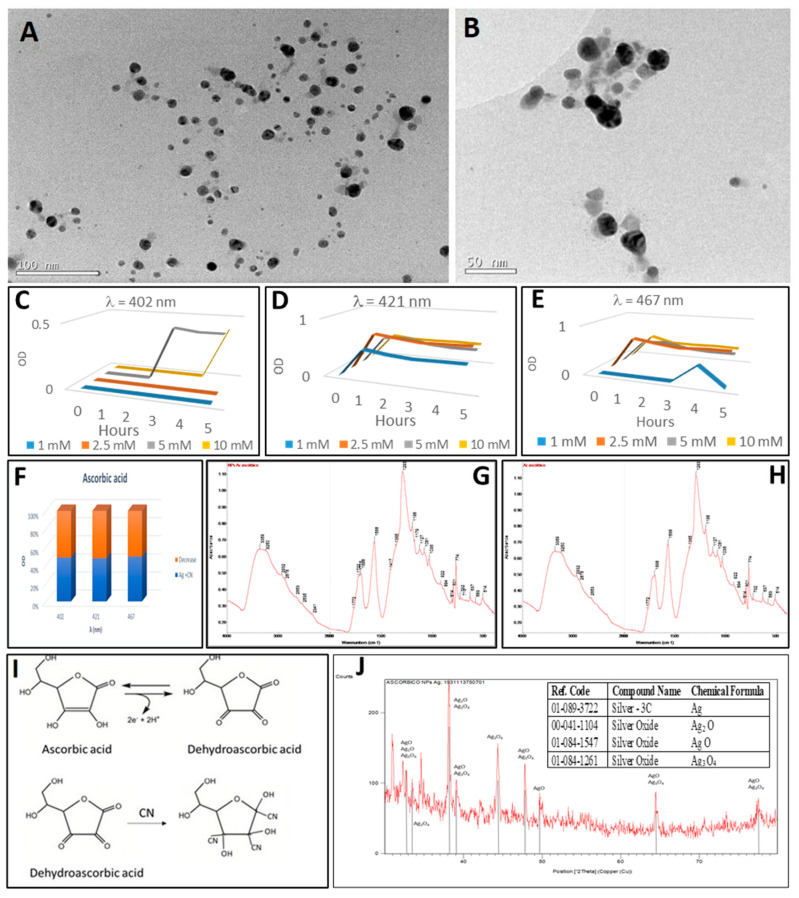
Analysis of silver nanoparticles synthesized with ascorbic acid. (**A**) TEM image of AgNPs. (**B**) image of AgNPs after treatment with cyanide. (**C**–**E**) Synthesis of silver nanoparticles by the reaction of different AgNO_3_ concentrations with ascorbic acid for 5 h (in one-hour intervals). (**C**) maximum at wavelength 402 nm (20 nm-diameter nanoparticles). (**D**) maximum at wavelength 421 nm (50 nm-diameter nanoparticles). (**E**) maximum at wavelength 467 nm (80 nm-diameter nanoparticles). (**F**) decrease of the OD after cyanide addition to silver nanoparticles. (**G**) Fourier transform infrared (FTIR) spectrum of ascorbic acid-derived nanoparticles. (**H**) FTIR spectrum of ascorbic acid. (**I**) Redox reaction of ascorbic acid to dehydroascorbic acid and effect of cyanide addition. (**J**) FTIR Scheme 3. in the insert, the table with the identifications. Peaks identifying the presence of silver oxides have been indicated in the XRD diffractogram.

The synthesis of silver nanoparticles with ascorbic acid is such an efficient system that, under optimal conditions, it can be used as a biosensor for ascorbic acid [82,83,84]. Ascorbic acid reacted immediately when added to the aqueous silver nitrate solution, with an instantaneous change in the colour of the solution from transparent to gold and a precipitation (Figure 2(A-4,B-4)). The yellow-gold colour of the reaction is caused by tiny light absorbing nanospheres with plasmon resonance peaks near 400 nm. The UV-visible absorption spectrum recorded during the reaction for five hours with varying amounts of silver nitrate showed three characteristic surface plasmon resonance bands of the silver nanoparticles at the wavelengths: 402 nm, 421 nm and 467 nm (Figure 6C–E). These nanoparticles had an average diameter of approximately 20 nm, as observed in the electron microscopy image (Figure 6A).

Ascorbic acid synthesised a high amount of 20 nm-size nanoparticles with plasmon resonance at λ = 402 nm. A relatively high concentration of silver nanoparticles of this size, 73.6 nM, was obtained. The concentration of silver nanoparticles with a diameter of 50 nm was 11.4 nM and that of bigger nanoparticles with a diameter of 80 nm only was 4.6 nM, as measured according to Beer–Lambert’s Law. The theoretical molar concentrations would be 3286 nM, 33 nM and 2.7 nM, respectively, as estimated assuming that the total conversion of the silver ions into silver nanoparticles of these sizes had taken place (Table 1). 

The FTIR signatures of the ascorbic acid and of the silver nanoparticles derived from ascorbic acid reduction were very similar (Figure 6G,H). For example, we identified a stretching vibration of the C=C bond and the peak of the enol-hydroxyl at 1568–1698 cm^−1^ and 1293 cm^−1^, respectively. The same occurred with the peaks at 3359 cm^−1^ and 3250 cm^−1^, which identify the O–H (hydroxyl) stretching bond. Also, there were two peaks at 1715 and 1722 cm^−1^ which correspond to the stretching of the C=O link. Peaks between 500 and 600 cm^−1^ are characteristic bands attributed to lattice vibration of silver oxide [46]. 

In the XRD analysis (Figure 6J), different oxidation states of silver were found: silver, silver oxide (Ag_2_O), silver (I,III) Oxide (AgO) and Ag_3_O_4_. The pattern with diffraction points at 2θ values of 38.14°, 44.34°, 64.44° and 77.48°, indexed to the planes (111), (200), (220) and (311) of the face-centred cubic structure (fcc), was compatible with the cubic phase of Ag. The average size of the silver crystalline particles as 40 nm was calculated with Scherrer’s equation.

The extinction spectrum of the silver nanoparticles after the addition of cyanide showed a reduction in the resonance plasmon that was observed as a decrease of the optical density by 50%. Specifically, this opacity was prominent at 402 nm, 421 nm and 467 nm wavelengths (Figure 6F). Therefore, it could be concluded that silver nanoparticles formed complexes with ascorbic acid, based on the FTIR spectrum analysis. The formation of these complexes produces fluorescence which is the basis for ascorbic acid biosensor systems using silver nanoparticles [85]. However, the molecule associated with the nanoparticles was dehydroascorbic acid (DHA; Figure 6I), which has three carbonyl groups in its structure. The possible synthesis mechanism of the silver nanoparticles could be explained by the oxidation of ascorbic acid to DHA. The silver nanoparticles would thus be capped with DHA [86]. This 1,2,3-tricarbonyl molecule is highly electrophilic and the polyhydroxyl structure is produced by irreversible hydrolysis of the ester bond [87]. The high reactivity of these silver-DHA nanoparticles has allowed the development of simple cyanide detection systems [88].

### 2.5. Synthesis of Silver Nanoparticles by 2-Hydroxybenzoic Acid

Salicylic acid or 2-hydroxybenzoic acid (C_7_H_6_O_3_) has a hydroxyl group and an acid group. The antioxidant capacity of this molecule is very limited. Although hydroxybenzoic acid can form chelates with metals, those formed with silver have very little stability [89]. 

**Figure 7:**
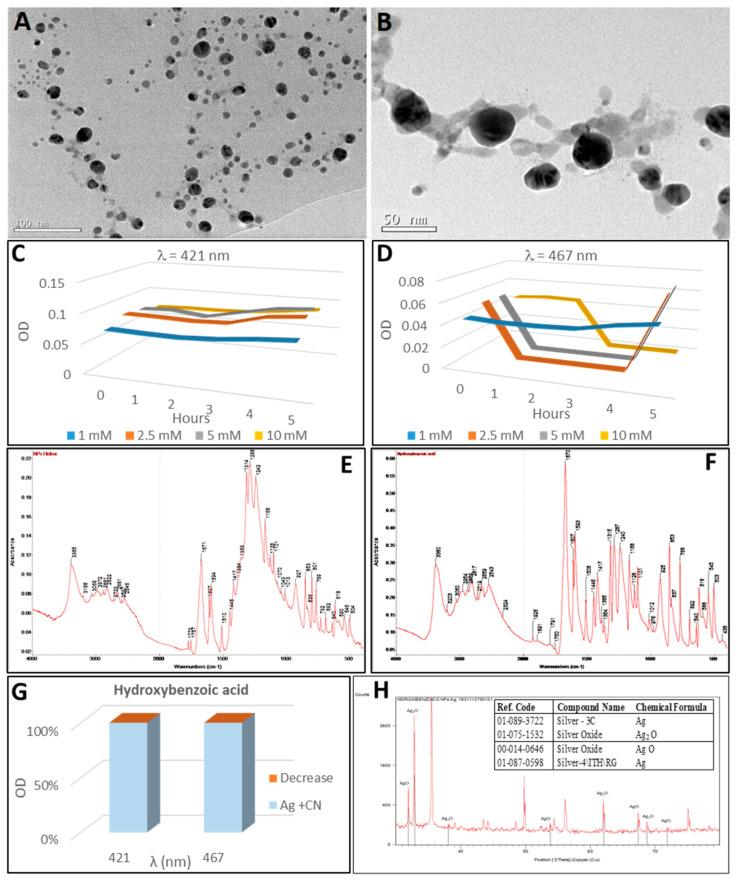
Analysis of silver nanoparticles synthesised with hydroxybenzoic acid. (**A**) TEM image of AgNPs prior to treatment with cyanide. (**B**) image of AgNPs after treatment with cyanide. (**C**,**D**) Synthesis of silver nanoparticles by the reaction of different AgNO_3_ concentrations with hydroxybenzoic acid for 5 h (in one-hour intervals). (**C**) maximum at wavelength 421 nm (50 nm diameter nanoparticles). (**D**) maximum at wavelength 467 nm (80 nm diameter nanoparticles). (**E**) FTIR spectrum of hydroxybenzoic acid-derived nanoparticles. (**F**) FTIR spectrum of hydroxybenzoic acid. (**G**) Relative reduction of OD after cyanide addition to silver nanoparticles. (**H**): XRD profile of the AgNPs obtained by the reaction of hydroxybenzoic acid with AgNO_3_; in the insert, the table with the identifications. Peaks identifying the presence of silver oxides have been indicated in the XRD diffractogram.

The addition of 2-hydroxybenzoic acid to a silver nitrate solution leads to a slow, inconspicuous reaction (Figure 2(A-5)) in which hardly any turbidity is apparent after five hours at 40 °C (Figure 2(B-5)). This slight reaction produces two small peaks in the UV-visible spectrum (Figure 7C,D) at the wavelengths of 421 nm (50 nm diameter) and 467 nm (80 nm diameter). The molar concentrations are 1.9 nM and 0.6 nM (Table 1) for the 50 nm and 80 nm nanoparticles, respectively. The synthesis of silver nanoparticles using salicylic acid requires an alkaline medium according to the literature [90,91]. Furthermore, m-hydroxybenzoic acid could promote the formation of stable AgNPs without additional capping agent [90]. However, the reaction should not depend on the presence of reagents outside the redox reaction itself. That is, only the pH-independent reaction, or in other words, the actual configuration redox potential, should be considered [92].

The synthesis of semi-spherical silver nanoparticles without forming aggregates, which were synthesized by silver reduction mediated by 2-hydroxybenzoic acid, was revealed by TEM imagery (Figure 7A). 

The FTIR spectra of hydroxybenzoic acid and of the silver nanoparticles synthesized by hydroxybenzoic acid-mediated reduction were very close (Figure 7E,F). For instance, peaks at 1240, 1287 and 1315 cm^−1^ were associated with the C–O bending, the peak at 1670 cm^−1^ corresponded to the carbonyl stretch and the 1240 cm^−1^ peak to phenol. The same applied to the peak at 3385 cm^−1^ from the O–H (hydroxyl) stretch bond. A peak approximately at 550 cm^−1^ is a characteristic band attributed to lattice vibration of silver oxide [86]. 

In the XRD analysis (Figure 7H), different oxidation states were also observed: silver, silver oxide (Ag_2_O) and silver (I,III) Oxide (AgO). The diffraction pattern at 2θ values of 38.15°, 44.33°, 64.48° and 77.52° could be indexed to the planes (111), (200), (220) and (311) of the face-centred cubic structure (fcc), which is compatible with the Ag phase. A 48 nm average size of the silver crystalline particles was estimated with the Scherrer’s equation.

Except for a slight increase in optical density of the UV-visible spectra after the addition of cyanide, variations for the wavelengths of 421 nm and 467 nm (Figure 7G) were not detected. These results could be related to the low reductive/antioxidant power of the hydroxybenzoic acid that was adhered to the silver nanoparticles.

### 2.6. Synthesis of Silver Nanoparticles by Caffeic Acid

Caffeic acid (3,4-dihydroxycinnamic acid) is a phenolic, hydroxycinnamic acid. It is a powerful antioxidant. In addition, caffeic acid has a high reducing power and is a metal chelator [93]. 

**Figure 8:**
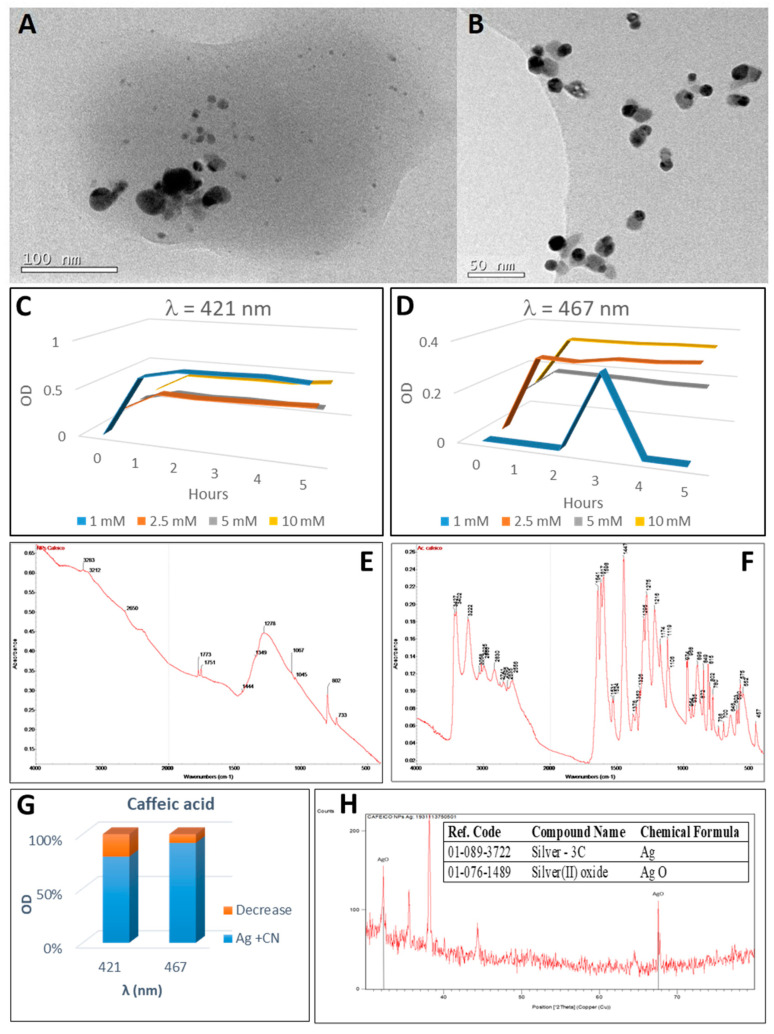
Analysis of silver nanoparticles synthesised with caffeic acid. (**A**) TEM image of AgNPs prior treatment with cyanide. (**B**) TEM image of AgNPs after treatment with cyanide. (**C**,**D**) Scheme 3. concentrations with caffeic acid for 5 h (in one-hour intervals). C: maximum at wavelength 421 nm (50 nm diameter nanoparticles). (**D**) maximum at wavelength 467 nm (80 nm diameter nanoparticles). (**E**,**F**) FTIR spectra of caffeic acid-derived nanoparticles (**E**) and caffeic acid (**F**). (**G**) decrease in OD after cyanide addition to silver nanoparticles. (**H**) XRD profile of the AgNPs obtained by the reaction of caffeic acid with AgNO_3_; in the insert, the table with the identifications. Peaks identifying the presence of silver oxides have been indicated in the XRD diffractogram.

Caffeic acid reacted immediately when added to the silver nitrate solution. The reaction became visible with the appearance of a dark grey colouration that contrasted with the transparency of the silver nitrate solution and the pale-yellow colour of the caffeic acid (Figure 2(A-6,B-6)). Silver nanoparticles could be synthesised quickly and simply by using caffeic acid as a reducing and stabilising agent in a similar way to that of gold nanoparticle synthesis [94]. In the UV-visible spectrum, two SPB peaks were detected at the wavelengths of 421 nm and 467 nm that would correspond to 50 nm and 80 nm diameter nanoparticles (Figure 8C,D). The most important synthesis was that of the 50 nm diameter nanoparticles, which started immediately for all concentrations tested, reaching high values of optical density. On the other hand, as the concentration of silver nitrate increases, the quantity of 50 nm-size nanoparticles decreases in favour of the appearance of the 80 nm diameter nanoparticles. The concentration of both varies between 13.5 nM (50 nm diameter nanoparticles) and 3.2 nM (80 nm diameter nanoparticles; Table 1). Spherical silver nanoparticles of different sizes and greyish colour can be visualised, synthesised by means of caffeic acid, and grey stuff surrounding the nanoparticles (Figure 8A).

When the FTIR spectrum of the nanoparticles synthesised with caffeic acid (Figure 8E) was analysed a wide and intense band can be observed in which the peaks at 2650 cm^−1^ (O–H stretching of carboxylic acid) and 3212–3283 cm^−1^ identifying the O–H stretching. The C=O bond appeared in the peaks at 1773-1751 cm^−1^ and the C–O bond at 1275 and 1278 cm^−1^ for nanoparticles and caffeic acid, respectively. The presence of silver oxide could be deduced by the presence of a broad band between 500 and 600 cm^−1^ [53]. The oxidized caffeic acid thus remained on the surface of the silver nanoparticles. Caffeic acid is well known for its strong adsorption on metal or metal oxide surfaces mainly due to the catechol functional group [95].

By means of the XRD analysis (Figure 8H), the following oxidation states were detected: 3C polytype of silver and silver oxide II (AgO). The pattern with diffraction points in 2θ values of 38.17°, 44.28°, 64.62° and 77.41° could be indexed to the planes (111), (200), (220) and (311) of the face-centred cubic structure (fcc), that is compatible with the Ag phase. The average size of the silver crystalline particles was 43 nm, as calculated by Scherrer’s equation.

When caffeic acid was used to synthesise silver nanoparticles from silver nitrate, the UV-visible spectrum remained virtually unchanged after the addition of cyanide. Only a small decrease in optical density at 421 nm and 467 nm (Figure 8G) can be observed. It is known that in caffeic acid, oxidation leads to the formation of the corresponding stable o-quinone through de-protonation of the initial semiquinone radical. The o-quinone can form an impediment or a steric effect around the silver nanoparticles that prevents the reaction [96]. This o-quinone in the proximity of the nanoparticles in the TEM image (Figure 8A,B) can be observed.

### 2.7. Synthesis of Silver Nanoparticles by Catechin

Catechin, DL-catechin, 2-(3,4-dihydroxyphenyl)chroman-3,5,7-triol or L-epicatechin, (C_15_H_14_O_6_) is a hydroxyflavan that has a flavan-3-ol skeleton. Catechin is soluble in water, although this solubility may increase with the addition of ethanol and the rise in temperature of the process [97]. Catechin has an antioxidant power that would allow its use as a phenolic standard in the quantification of reducing power in biological foods and fluids [98,99]. In addition to its antioxidant capacity, it has the capacity to chelate metals [100]. Furthermore, it has been demonstrated that catechin is a much more effective antioxidant in a complex with metals than in its free form [101]. All these properties have determined the ability to use the synthesis of silver nanoparticles by catechin to develop sensors [102]. Catechin has been extensively studied for its therapeutic potential and AgNPs synthesized by catechin showed significant antibacterial, anticancer and protein-binding properties [103].

**Figure 9:**
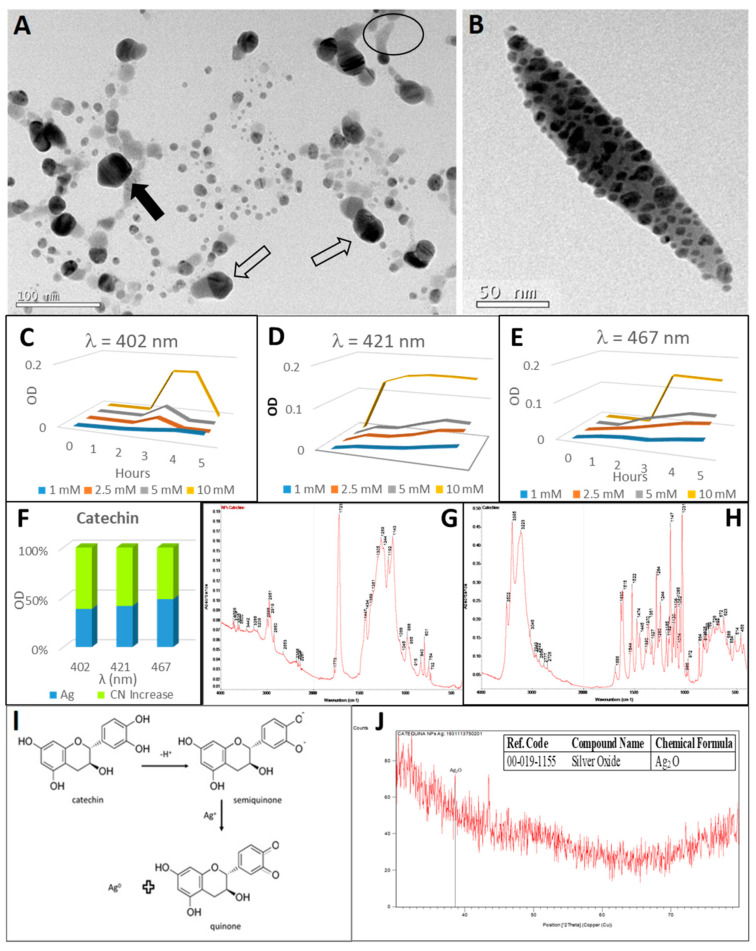
Analysis of catechin-synthesised silver nanoparticles. (**A**) TEM image of pentagonal (solid arrow) and polyhedral (hollow arrow) AgNPs prior to cyanide treatment. (**B**) TEM image of AgNPs after treatment with cyanide. (**C**–**E**) Synthesis of silver nanoparticles by the reaction of different AgNO_3_ concentrations with catechin for 5 h (in one-hour intervals). (**C**) Maximum at 402 nm wavelength (20 nm diameter nanoparticles); (**D**) 421 nm (50 nm diameter nanoparticles) and (**E**) maximum at wavelength 467 nm (80 nm diameter nanoparticles). (**F**) Relative increase of OD after cyanide addition to silver nanoparticles. (**G**,**H**) FTIR spectra of catechin-derived nanoparticles (**G**) and catechin (**H**). (**I**) Synthesis reaction of silver nanoparticles with catechin mediated by semiquinone and quinone formation. (**J**) XRD profile of the AgNPs obtained by the reaction of the catechin with AgNO_3_; in the insert, the table with the identifications. Peaks identifying the presence of silver oxides have been indicated in the XRD diffractogram.

The reaction of catechin with silver nitrate was observed after a period of more than one hour (Figure 2(A-7,B-7)). The reaction was obvious by the appearance of a dark golden colour and the formation of a precipitate. The changes of colour from white turbid into dark brown is caused by the oxidation of catechin [104] to create another compound of semiquinone and quinone [105]. Three SPBs corresponding to the wavelengths 402 nm (20 nm diameter), 421 nm (50 nm diameter) and 467 nm (80 nm diameter) after five hours of reaction (Figure 9C–E) have been observed. The higher concentration of silver nitrate in the reaction favoured the synthesis of these nanoparticles, being the 50 nm diameter ones the first to be synthesised. After five hours of reaction the highest molar concentration of nanoparticles (27.3 nM) was reached for 20 nm-size particles (Table 1). 

High-resolution transmission electron microscopy revealed the synthesis of silver nanoparticles thanks to catechin (Figure 9A). Furthermore, the capping ability of catechin could also be observed in the same image. A dark grey halo appeared in the vicinity of the nanoparticles (surrounded by a circle in the image). It can be inferred that the mixed crystalline and amorphous nature of the catechin-mediated AgNPs from the XRD profile. AgNPs had pentagonal (Figure 9A, solid arrow) and polyhedral (Figure 9A, hollow arrow) forms. Smaller spherical nanoparticles (20 nm) were putative “cores” for the subsequent synthesis of the 50 nm diameter pentagonal-based polyhedral particles [106]. The pentagonal arrangement in metal nanoparticles is quite well known. The synthesis of these nanoparticles is thermodynamically favoured, mainly in very small sizes (less than 20 nm). As the particle size increases, the structure evolves into an fcc-type particle, such as a cuboctahedron. However, for larger particles as in this case, the kinetics of growth starting with the size, shape and structure of the nuclei, should play a critical role in determining the shape of the final product [107]. 

The peaks at 3395 cm^−1^ and 3223 cm^−1^ in the catechin FTIR spectrum (Figure 9H) revealed the O–H stretching of the alcohol group. In addition, the peaks at 1147 and 1031 cm^−1^ corresponded to the C–O bending. On the other hand, an intense and narrow peak at 1723 cm^−1^ was observed, indicating the C=O stretching bond in the nanoparticle FTIR spectrum (Figure 9G), and also a wide band with peaks at 1298-1143 cm^−1^ corresponding to the C–O bending, and a peak at 2951 cm^−1^ of the C–H stretching bond. The peak at 801 cm^−1^ represented the C–H bending in the aromatic ring. Thus, an association of the quinone resulting from the oxidation of catechin could take place in the process of formation of silver nanoparticles from silver nitrate. This quinone would be identified in the electron microscopy image (Figure 9I, surrounded by a circle). A peak near 500 cm^−1^ indicates the presence of silver oxide bond [108]. 

Only the presence of Ag_2_O silver oxide through XRD analysis (Figure 9J) was observed, probably because the amorphous quinone’s signature masked other compounds [109]. The Ag_2_O oxidative form of silver is easily obtained [7].

When cyanide was added to the suspension of catechin antioxidant-promoted silver nanoparticles, the optical density of the SPB increased (Figure 4). This increase varied from 40% for 467 nm wavelength to 60% for the wavelengths 402 nm and 421 nm. It is possible this could be attributed to the interaction between tannin and cyanide [110], although more studies will be necessary in this respect.

The silver nanoparticles were immersed in a grey matrix (TEM image, Figure 9B), producing the appearance of heterogeneous clumps of nanoparticles surrounded by the matrix. The formation of this matrix could be attributed to the decrease in stabilising capacity of catechin during the reaction, which allows the formation of a high molecular weight polymer, probably a condensed tannin consisting of catechin units. Similar matrices have previously been described through TEM imagery when nanoparticles were synthesised with tannins [111]. Also, other authors have described the same pattern of silver nanoparticle dispersion in the polymer network [112].

### 2.8. Synthesis of Silver Nanoparticles by Scopoletin

Scopoletin (synonyms: gelseminic acid, 6-methylesculetin, 7-hydroxy-6-methoxy-2H-chromen-2-one) is a hydroxycoumarin, an umbelliferone that has a methoxy substitute at position 6 (C_10_H_8_O_4_). It is slightly soluble in water (2.35 g L^−1^). The antioxidant activity shown by scopoletin is very weak [113]. In addition, its capacity as a chelator is also low [114].

**Figure 10:**
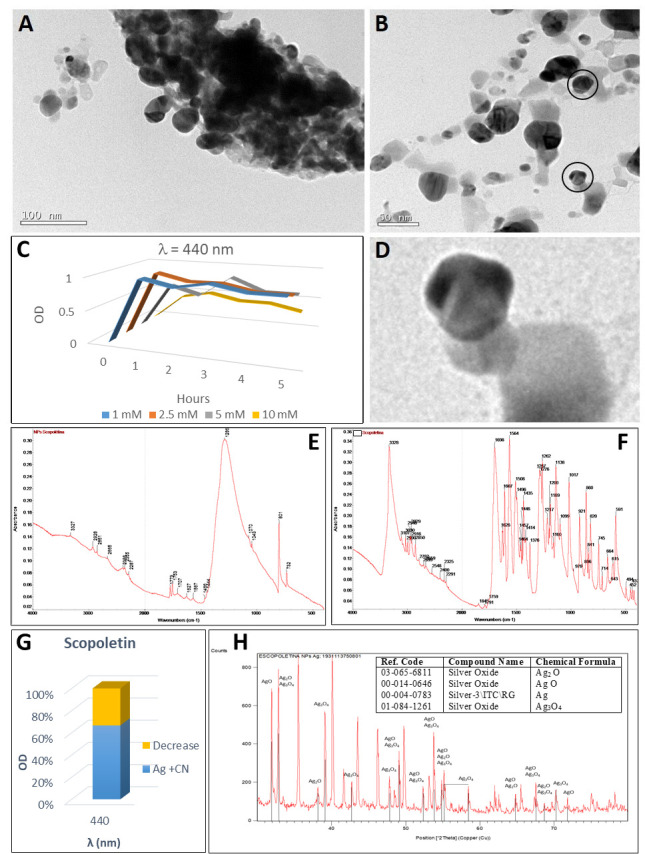
Analysis of the silver nanoparticles synthesised with scopoletin. (**A**) TEM image of AgNPs prior to cyanide treatment. (**B**) TEM image of AgNPs after cyanide treatment. (**C**) Synthesis of silver nanoparticles by the reaction of different AgNO_3_ concentrations with scopoletin for 5 h (in one-hour intervals). Maximum at 440 nm wavelength (70 nm diameter nanoparticles). (**D**) Detail of nanoparticle aggregation, with cookie-like form. (**E**,**F**) FTIR spectra of scopoletin-derived nanoparticles (**E**) and scopoletin (**F**). (**G**) decrease in OD after cyanide addition to silver nanoparticles. (**H**) XRD profile of AgNPs obtained by reaction of scopoletin with AgNO_3_; in the insert, the table with the identifications. Peaks identifying the presence of silver oxides have been indicated in the XRD diffractogram.

Scopoletin did not react immediately with silver nitrate (Figure 2(A-9)) but after one hour of reaction at 40 °C (Figure 2(B-9)) the solution changed from transparent to greyish green. The UV-visible spectrum showed only one SPB peak at 440 nm which decreased when the highest nitrate concentration of our experiment (10 mM) was applied, possibly because of nanoparticle precipitation. A 70 nm silver nanoparticle diameter from the resonance plasmon wavelength (440 nm) was estimated. The concentration of these nanoparticles was 11.1 nM (Table 1), about one tenth of what could have achieved if 100% of the silver ions in the solution would have converted to nanoparticles (100 nM). The silver nanoparticle synthesis mediated by scopoletin reduction formed a precipitate (Figure 10A).

FTIR spectra of both scopoletin and scopoletin-derived nanoparticles (Figure 10E,F) showed a peak at 3327–3328 cm^−1^, corresponding to the C–H stretching. However, the peak in the scopoletin standard was well defined, while the band was broader in the case of nanoparticles. Also, bands corresponding to aromatic C=C stretches (between 1500 and 1600 cm^−1^) and C=O stretching (close to 1700 cm^−1^) were identified. An intense peak at 1286 cm^−1^ revealed the C–O stretching. The peak at 801 cm^−1^ was associated to a C–H bending (675–1000 cm^−1^) which corresponds to the C–H stretching at 2851–2928 cm^−1^. These bands represented the vibrations of the C-H link in the aromatic ring.

Also, different silver oxidation states in the XRD analysis (Figure 10H) were observed: silver, silver oxides (Ag_2_O, Ag_2_O_3_), and silver (I,III) oxide (AgO). The pattern with diffraction points at 2θ values of 38.15°, 44.33°, 64.52° and 77.52° could be indexed to the planes (111), (200), (220) and (311) of the face-centred cubic structure (fcc), which is compatible with the cubic phase of Ag. The average size of the silver crystalline particles was 63 nm, as calculated by Scherrer’s equation.

The addition of cyanide barely decreased the optical density of the absorption spectra (Figure 10G). Dispersed nanoparticles with heterogeneous shapes can be observed in the TEM image (Figure 10B). Greater, cookie-like NPs were clusters of nanoparticles (Figure 10D), in agreement with the description of cookie-like NPs by other authors [115].

## 3. Material and Methods

### 3.1. Materials

For the synthesis of silver nanoparticles, silver nitrate (AgNO_3_) and the following reducing agents were used (Figure 1): quercetin, β-carotene, gallic acid, ascorbic acid, hydroxybenzoic acid, caffeic acid, catechin and scopoletin. All reagents were obtained from Sigma-Aldrich Quimica SL, Madrid, Spain. All reagents were used directly.

### 3.2. Preparation of Chemical Solutions

The stock solutions were all prepared using distilled water, 2 M silver nitrate solution and 100 µM solutions of each antioxidant. The two less hydrophilic antioxidants (quercetin and β-carotene) were kept in constant agitation. 

### 3.3. Preparation of Nanoparticles

For the preparation of the reaction mixture, the silver nitrate solution was mixed with the appropriate volume of solution of each antioxidant to achieve the following final concentrations of silver nitrate in the final volume: 1 mM, 2.5 mM, 5 mM and 10 mM with a 100 µM concentration of the antioxidant. The reduction reaction was carried out at 40 °C for 5 h. Constant stirring was used to prepare and use the antioxidant solutions.

### 3.4. Characterization of Nanoparticles

#### 3.4.1. Spectrophotometry

The UV-visible absorption spectra of the Ag nanoparticle solutions were analysed in the wavelength range of 300 to 500 nm using a UV-visible spectrophotometer. The results are presented as bar graphs when the wavelength of the resonance plasmon can be determined, otherwise only the spectrum is presented. 

#### 3.4.2. Transmission Electron Microscopy (TEM) 

The nanoparticles were dispersed in 1 mL of water. The TEM grids were prepared by placing a drop of the particle solution on a carbon-coated copper grid and drying it at room temperature. TEM was performed with a JEOL2100 microscope at the Centro Nacional de Microscopía Electrónica, Av. Complutense s/n, 28040 Madrid.

#### 3.4.3. X-ray Powder Diffraction

The X-ray powder diffraction (XRD) profiles were obtained from an Empyrean Cu LFF X-ray diffractometer with a 2 theta range from 2 to 80. The analyses were performed at the Research Support Centre (CAI) for X-Ray Diffraction of the Universidad Complutense de Madrid, Ciudad Universitaria s/n, 28040 Madrid. The resulting diffraction patterns (measured with XRD) were compared with reference patterns from the database of diffraction patterns (International Centre for Diffraction Data; ICDD database).

The average size of the nanoparticles was calculated according to Scherrer’s Equation (1) [116,117]:

D(nm) = kλ/βcosϴ(1)

Definition of terms:

D = the average crystallite size (nm),

k = Scherrer constant, 

λ = X-ray wavelength,

β= full width at the half maximum of the peak (FWHM). This has to be converted to radians

θ = angle of diffraction.

Evaluation of the XRD analysis was used to identify the nature of the nanoparticles formed. First, the standard 2θ values for silver nanoparticles in the form of nanocrystals given in the literature were indexed to the planes (111), (200), (220), (311) and (222). Then, the presence of other peaks from 20 to 80 in the XRD diffractogram indicated the presence of silver oxides. The identified phases for silver oxides were indicated and the identified patterns list were inserted as a table in the graph.

#### 3.4.4. Fourier-Transform Infrared Spectroscopy 

The Fourier-transform infrared (FTIR) spectroscopy measurements were made with JASCO (FT/IR-6200) spectrophotometer at the CAI of Physical and Chemical Techniques, Spectroscopy and Correlation Unit, of the Complutense University of Madrid, Plaza de Ciencias, 2, 28040 Madrid. The analysis of the results was developed according to the tables included in [118]. 

#### 3.4.5. Oxidation of AgNPs in the Presence of Cyanide

The interaction between aqueous silver nanoparticles (AgNP) and cyanide ions was studied using UV-visible absorption. The method is based on the oxidation of AgNP with dissolved oxygen in the presence of cyanide ions, which produces a decrease in the intensity of the surface plasmon resonance (SPB) absorption band of AgNPs [119]. This phenomenon occurs as a consequence of the formation of the colourless Ag(CN)_2_ complex according to the following reaction: Ag + 2CN^−^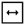 Ag(CN)_2_^−^ + e^−^

The absorption spectrum between 300 and 500 nm wavelengths was analysed by adding an equal volume of each antioxidant to the silver nitrate (2 M) stock solution and compared with the spectrum when half a volume of a 5 mM cyanide solution was added. 

#### 3.4.6. Estimation of the Concentration of Silver Nanoparticles

Two different methods were used to estimate the concentration of silver nanoparticles synthesized by each of the reducing agents:

—From the UV-visible spectrum and the mean nanoparticle size determined by TEM according to Paramelle et al. [120]. The molar concentration of silver nanoparticles (mol L^−1^) is calculated by applying Beer-Lambert’s Law Equation (2):

C = A/ὲ(2)

where C is the molar concentration, A is the maximum absorbance and ὲ is the molar extinction coefficient.

—By calculating the molar concentration according to Kalishwaralal et al. [121]. According to the formula proposed by Equation (3) Liu et al. [122]:

C = N_T_/N · V · N_A_ (3)

where C is the molar concentration of nanoparticles, N_T_ is the total number of silver atoms added as AgNO_3_, N is the number of atoms of each nanoparticle, V is the volume of the synthesis reaction in L and N_A_ is the number of Avogadro (6.023 × 10^23^).

## 4. Conclusions

Silver-containing nanoparticles were synthesized using synthetic plant extracts. The basis for this was the antioxidant activity of these extracts thanks to the numerous secondary metabolites synthesized by plants. The antioxidants analysed in this study (quercetin, β-carotene, gallic acid, ascorbic acid, hydroxybenzoic acid, caffeic acid, catechin and scopoletin) showed different capacities of synthesis, different shapes and sizes, and different aggregations and presence of capping agents. The resulting nanoparticles showed different reactivity associated with all these characteristics. Among our results, the micelle-like structures obtained with quercetin, the triangular nanoparticles and polyhedral forms, both with truncated corners synthesized with β-carotene and the pentagonal nanoparticles synthesized with catechin, stood out. Differences in reactivity were observed among the synthesized nanoparticles, from the minimum level detected for the nanoparticles synthesized with hydroxybenzoic acid to the most reactive nanoparticles synthesized with ascorbic acid. Two exceptions were observed for this method of studying the reactivity of nanoparticles with cyanide. The first one concerned nanoparticles synthesized with gallic acid, which could be associated to the presence of Ag^+^ (partially oxidized silver) on the surface of the nanoparticles. The second one referred to nanoparticles synthesized with catechin. In the latter, the presence of tannins derived from the polymerization of catechin during the synthesis reaction of silver nanoparticles could be the cause, as these tannins could act as inhibitory agents of the reaction of the nanoparticles with cyanide. This study on the role of each of these metabolites on the synthesis of silver nanoparticles is a first step to optimise the methodology of green synthesis.

## Figures and Tables

**Table 1:** Concentration (nM) of nanoparticles of different sizes (20, 50, 70 and 80 nm), synthesised by various antioxidants (gallic acid, ascorbic acid, hydroxybenzoic acid, caffeic acid, catechin and scopoletin). The concentrations were calculated from Beer-Lambert’s Law (according to Paramelle et al. 2013). Theoretical: theoretical molar concentration, assuming the total conversion of silver ions into silver nanoparticles (according to Kalishwaralal et al. 2010).

	Concentration (nM)			
Antioxidant	20 nm	50 nm	70 nm	80 nm
Gallic acid	-	-	30	-
Ascorbic acid	73.6	11.4	-	4.6
Hydroxybenzoic acid	-	1.9	-	0.6
Caffeic acid	-	13.5	-	3.2
Catechin	27.3	2.9	-	1.1
Scopoletin	-	-	11.1	-
Theoretical	4000	260	100	53
